# Prevalence and molecular detection of *Theileria* in ixodid ticks in central and southern Shanxi, China

**DOI:** 10.1016/j.parepi.2026.e00533

**Published:** 2026-08-14

**Authors:** Jia Cui, Fengping Wang, Huaxiang Rao, Bosi Dang, Yuyao Dou, Yiping Liu, Jingrong Niu, Liqing Wang, Changxin Wu, Juan Yu

**Affiliations:** a School of Basic Medical Sciences, Changzhi Medical College, Changzhi 046000, China; b School of Public Health, Changzhi Medical College, Changzhi 046000, China; c Key Laboratory of Cellular Physiology, Ministry of Education, and the Department of Physiology, Shanxi Medical University, Taiyuan 030012, China; d Institutes of Biomedical Sciences, Shanxi University, Taiyuan 030006, China

**Keywords:** *Theileria luwenshuni*, *Theileria orientalis*, China, Ixodid ticks, Phylogeographic analysis

## Abstract

**Background:**

*Theileria*, an obligate intracellular parasitic protozoan transmitted primarily by ixodid ticks, poses a significant threat to animal health and livestock productivity. This study conducted a comprehensive investigation into the prevalence and genetic diversity of *Theileria* species in ixodid ticks in central and southern Shanxi Province, China.

**Methods:**

First, a certain number of tick samples were collected, including 102 sheep-infesting ticks from Baitupo Village and Wangjiazhuang Village in Yangquan City, Shanxi Province, China, and a total of 60 cattle-infesting ticks from Daxigou Village in Yuncheng City and Matian Town in Jinzhong City, Shanxi Province. Then, genomic DNA was extracted from tick samples, followed by conventional PCR targeting a fragment of the *18S rRNA* gene for the detection of *Theileria*. All positive amplicons were subsequently sent for Sanger sequencing. The obtained nucleotide sequences were subjected to phylogenetic analysis along with reference sequences retrieved from NCBI GenBank using MEGA-X software. DnaSP, Arlequin and PopART software were employed to conduct haplotype-based geographical evolution analysis for the same species of *Theileria.*

**Results:**

Molecular screening revealed an overall average *Theileria* infection prevalence of 67.28% (109/162) across the surveyed samples. The positive rates varied markedly by sampling site: 77.42% (48/62) in Baitupo Village, 47.50% (19/40) in Wangjiazhuang Village, 61.90% (13/21) in Daxigou Village, and 74.36% (29/39) in Matian Town. The infection rates of *Theileria* showed statistically significant differences among different regions (*χ*^2^ = 9.497, *P* = 0.023). Among these, forty sequences from positive sheep-infesting ticks for *Theileria* infection were identified as *Theileria luwenshuni*. The 11 sequences from positive cattle-infesting ticks were classified as *Theileria orientalis.* Phylogenetic analysis displayed that sequences of *T. luwenshuni* had the closest genetic relationship with strains previously identified in Changzhi, Shanxi, China (GenBank: OR999605). Meanwhile, *T. orientalis* sequences clustered together with the Chinese *T. orientalis* reference strain (GenBank: OQ507242.1). The phylogeographic analysis revealed that all *T. luwenshuni* isolates examined in this study belonged to a single conserved haplotype, while six divergent haplotypes were identified among *T. orientalis* sequences.

**Conclusions:**

Our study revealed a high prevalence of *Theileria* infection among ixodid ticks in central and southern Shanxi, China, underscoring an urgent demand for targeted intervention strategies to limit the transmission of theileriosis and alleviate its rising risks to veterinary and public health.

## Graphical abstract

**Figure:**
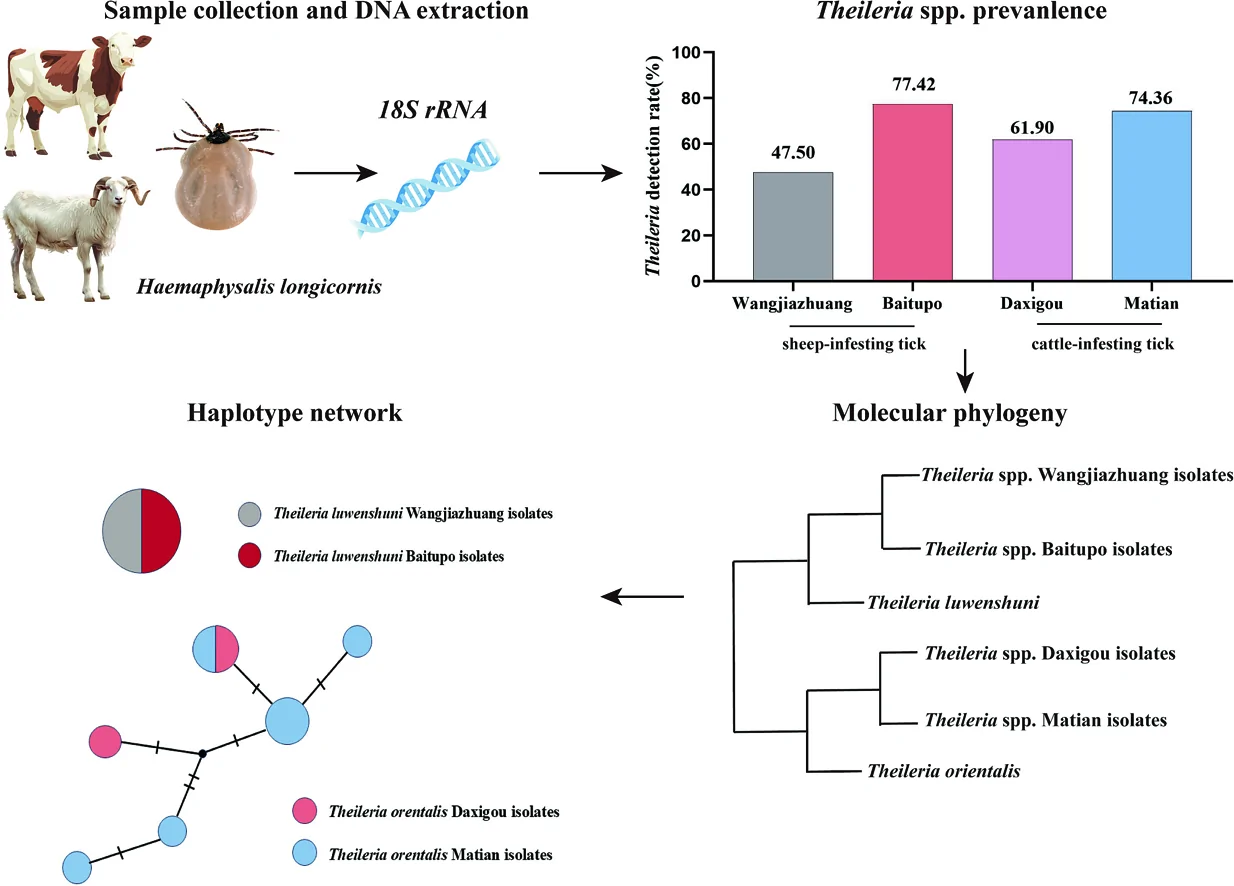
Prevalence and molecular detection of *Theileria* in ixodid ticks in central and southern Shanxi, China

## 1 Background

Ticks are key vectors for numerous zoonotic parasites and pathogens, including *Borrelia burgdorferi*, *Rickettsia*, *Coxiella*, *Babesia*, *Theileria*, Dabie bandavirus, Crimean-Congo hemorrhagic fever virus, forest encephalitis virus and others (Maldonado-Ruiz, 2024; Moming et al., 2024). Among them, *Theileria* are obligate intracellular apicomplexan parasites that infect tick salivary gland cells and vertebrate host erythrocytes (Woods et al., 2021). Beyond functioning as *Theileria* vectors, ticks provide an obligate biological niche necessary for the parasite to fulfil its multi-stage developmental cycle (Scoles and Ueti, 2015). Different tick species often display distinct host specificity toward certain *Theileria* taxa, resulting in intricate co-evolved host-parasite relationships that shape the ecological characteristics and evolutionary pathways of disease (Tajeri et al., 2021). When animals are infected with *Theileria*, the infection may cause anemia, pyrexia, lymphadenopathy, and even death (Bishop et al., 2004). As theileriosis continues to spread, it poses a serious and ongoing threat to the global livestock industry. Therefore, precise detection of tick-borne *Theileria* infections carries great importance for epidemiological research and disease management.

*Theileria* is primarily prevalent in tropical and subtropical regions worldwide, with northeastern and northwestern China also being severely affected area (Sivakumar et al., 2014; Jia et al., 2020). Longitude, latitude, precipitation, temperature, humidity, and altitude may influence the distribution of *Theileria* (Chen et al., 2022). Situated in northern China, Shanxi Province possesses a typical continental monsoon climate. Its undulating landscape of mountains and basins provides favorable habitats for arthropod vectors, thereby potentially enhancing the transmission dynamics of theileriosis. In China, *Theileria annulata* and *Theileria sergenti* are widely prevalent, followed by *Theileria luwenshuni*, *Theileria sinensis*, *Theileria buffeli*, *Theileria orientalis*, *Theileria ovis*, *Theileria uilenbergi* (Junlong et al., 2015)*.* Currently, the diagnosis of *Theileria* infection primarily relies on molecular and serological testing. Cross-reactivity among closely related species restricts the application of indirect fluorescent antibody tests (IFAT) (Bakheit et al., 2007). Meanwhile, The application of various molecular biology techniques has enhanced both the sensitivity and specificity for detection *Theileria* spp. (Maharana et al., 2016; Nangru et al., 2022).

The present study was conducted to investigate the presence of *Theileria* in ticks via molecular biology methods (PCR and sequencing), and further characterize their prevalence and genetic diversity. The findings will address gaps in surveillance for tick-borne *Theileria* in central and southern Shanxi, China, and provide data support for developing targeted disease prevention and control strategies.

## 2 Methods

### 2.1 Ticks collection

**Fig. 1:**
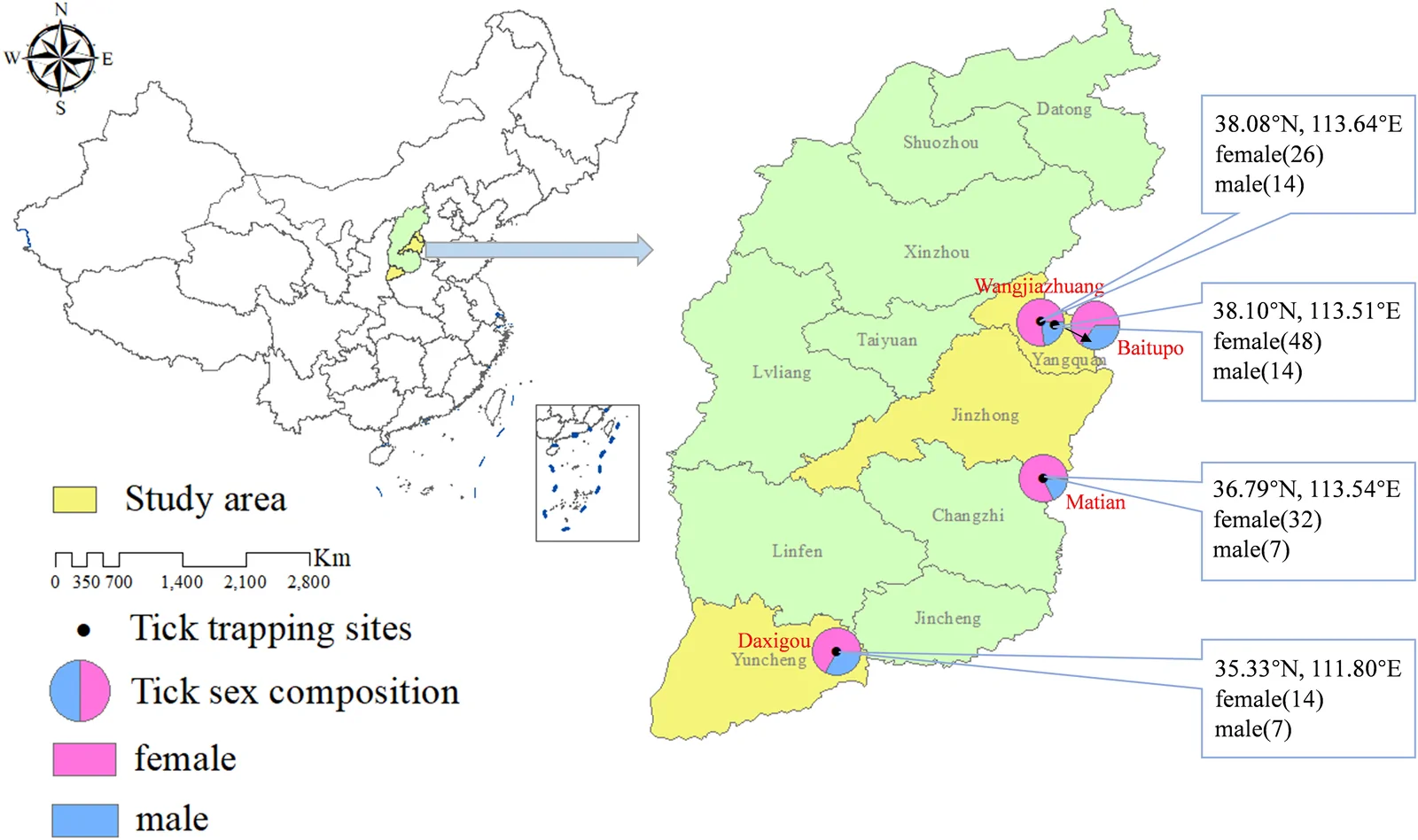
Geographical Distribution of Ticks Collected in Shanxi, China. This map illustrates the geographical distribution of sampling sites within China. Each pie chart corresponds to one sampling site, and the segments within the chart illustrate the proportional composition of male and female ticks. This map was created in ArcGIS 10.8 with base map data sourced from the National Platform for Common GeoSpatial Information Services (https://www.tianditu.gov.cn/). The data are solely for illustrative research purposes and are available free of charge.

From June to September 2024, ticks were collected manually with forceps from the ears, abdomens and other body parts of host animals. The tick sampling sites were randomly choosen from four locations across three cities in central and southern Shanxi Province, China: Wangjiazhuang Village (Suburban District, Yangquan City), Baitupo Village (Yu County, Yangquan City), Daxigou Village (Yuanqu County, Yuncheng City) and Matian Town (Zuoquan County, Jinzhong City). The four regions are located at latitudes 35.33° to 38.10° north and longitudes 111.80° to 113.64°east, and the geographical distribution is shown in Fig. 1. Adult ticks were collected from sheep in Wangjiazhuang Village (*n* = 40) and Baitupo Village (*n* = 62). Meanwhile, all ticks sampled in Daxigou Village (*n* = 21) and Matian Town (*n* = 39) were cattle-infesting ticks. This study was approved by the Ethics Committee of Changzhi Medical College (No: DW2023011).

Ticks were preliminarily classified based on their morphological characteristics under a microscope. Subsequently, representative samples were selected and subjected to mitochondrial cytochrome *c* oxidase subunit I (COI) gene sequencing for molecular identification. Primer sequences for COI gene amplification were as follows: forward, 5′-GGT CAA CAA ATC ATA AAG ATA TTG G-3′; reverse, 5′-TAA ACT TCA GGG TGA CCA AAA AAT CA-3′. The amplicon was 683 base pairs in length. Ticks were stored in sterile tubes at −80 °C from morphological identification to molecular analysis. For each sampling site, one farm was enrolled in the investigation, with no fewer than five animals screened per farm. Three to five ticks were harvested from each animal. The screening criterion for tick samples required intact tick bodies and fully preserved mouthparts.

### 2.2 *Theileria* detection and sequencing

Ticks were washed 3–5 times in distilled water, with each soaking lasting 5 min. Subsequently, the specimens were gently air-dried under sterile conditions at room temperature to preserve their integrity for further analysis. Each tick was processed with the TIANamp Micro DNA Kit according to the manufacturer's instructions (TIANGEN Biotech (Beijing) Co., Ltd., China) to isolate genomic DNA. To amplify the target *18S rRNA* gene of *Theileria*, PCR was conducted using specific primers (Forward: 5´-GTC TTG TAA TTG GAA TGA TGG-3′, Reverse: 5′-TAG TTT ATG GTT AGG ACT ACG-3′) in a 20 μL reaction system containing EasyTaq SuperMix (TransGen Biotech). Additionally, for cattle-infesting ticks, we further perform amplification and identification based on their Major piroplasm surface protein (*MPSP*) gene of *T. sinensis* and *T. orientalis*, with primer sequences referenced from Li et al. (Li et al., 2015). The amplification was performed under the following conditions: one cycle for 10 min at 94 °C; 35 cycles for 30 s at 94 °C, 30 s at 55 °C, and 45 s at 72 °C; and a final extension for 10 min at 72 °C. Next, the PCR products were identified by 1.5% agarose gel electrophoresis, and then sent to Sangon Biotech (Shanghai, China) for sequencing.

### 2.3 Phylogenetic analysis

The obtained sequencing data were aligned using BLAST against the reference *Theileria 18S rRNA* gene sequences available in GenBank, followed by deposition in the NCBI database. Phylogenetic analysis was performed on sequences from this study and homologous *18S rRNA* reference sequences retrieved from NCBI database. A phylogenetic tree was constructed based on the sequences generated in this study and homologous reference *18S rRNA* sequences. All nucleotide sequences were first aligned using ClustalW for maximum positional homology. Phylogenetic reconstruction was then performed under the neighbor-joining (NJ) framework to infer evolutionary history, with nodal confidence quantified by 500 bootstrap iterations in MEGA-X.

### 2.4 Haplotype analysis

The analysis of nucleotide and haplotype diversity was performed using DnaSP 6.12.03 by identifying conserved sequences and polymorphic sites. Key output metrics included the number of sequences (n), the number of polymorphic sites (S), the number of haplotypes (h), nucleotide diversity (*π*), average number of nucleotide differences (*κ*), haplotype diversity (Hd) and associated variance values. Subsequently, Arlequin 3.5.2.2 was employed to cluster similar sequences, outputting a matrix for haplotype network construction. This matrix was imported into the PopART 1.7, and the Median-Joining Network (MJN) algorithm was applied to optimize the resolved *Theileria* haplotypes.

### 2.5 Statistical analysis

The positivity rates of *Theileria* infection across distinct tick sampling sites and parasitic hosts were compared using Chi-Square test to assess potential epidemiological associations. All statistical analyses were conducted in SPSS 22.0 (SPSS, Inc., Chicago, IL, USA), with *P*-value <0.05 considered statistically significant.

## 3 Results

### 3.1 Ticks collection

In 2024, a total of 162 ticks were collected using the host-attached collection method in the central and southern region of Shanxi Province, China. Preliminary morphological identification of tick species and developmental stages was carried out under a stereomicroscope using published taxonomic keys (Barker and Walker, 2014). All specimens were identified as adult *Haemaphysalis longicornis*. COI gene amplification and sequencing were performed on 23 selected representative tick samples, and all individuals were confirmed to be *Haemaphysalis longicornis*, which is the dominant Ixodidae species in north China.

### 3.2 Infections of *Theileria* species

To investigate infection with *Theileria* spp. in ticks in central and southern Shanxi, 102 sheep-infesting and 60 cattle-infesting ticks from four sampling sites were subjected to *Theileria* detection. The detection of *Theileria* was performed using a previously described species-specific PCR method targeting the partial *18S rRNA* gene (429 bp). From Table 1, the infection rates of *Theileria* in the four regions-Wangjiazhuang Village, Baitupo Village, Daxigou Village, and Matian Town-were up to 47.50%, 77.42%, 61.90%, and 74.36%, respectively. A representative nucleic acid gel image was provided in Fig. S1. Moreover, the study revealed geographical variations in *Theileria* infection rates across the four surveyed regions (*χ*^2^ = 9.497, *P* = 0.023). Notably, Baitupo Village had the highest prevalence (77.42%), significantly higher than Wangjiazhuang Village (47.5%). Aross the four sampling locations, the total *Theileria* prevalence of female ticks reached 86.67%, which was significantly higher than the 11.90% prevalence observed in male ticks (Table S1) (*χ*^2^ = 30.446, *P* < 0.001). As shown in Table 2, the *Theileria* infection rate in cattle-infesting ticks (70.00%) was numerically higher than that in sheep-infesting ticks (65.69%), but this difference was not statistically significant (*χ*^2^ = 0.319, *P* = 0.572).

**Table 1:** The polymerase chain reactions (PCR) diagnose of *Theileria* in different sites.

Sampling sites	Tick types	No.captured	No.PCR positive	Positivity rate (95% CI, %)
Wangjiazhuang Village, Suburban District, Yangquan City	female	26	19	73.08 (53.92, 86.30)
	male	14	0	0.00 (0.00, 21.53)
	total	40	19	47.50 (32.94, 62.50)
Baitupo village, Yu County, Yangquan City	female	48	45	93.75 (83.17, 97.85)
	male	14	3	21.43 (7.57, 47.59)
	total	62	48	77.42 (65.60, 86.04)
Daxigou Village, Yuanqu County, Yuncheng City	female	14	12	85.71 (60.06, 97.46)
	male	7	1	14.29 (0.73, 51.31)
	total	21	13	61.90 (40.88, 79.25)
Matian Town, Zuoquan County, Jinzhong City	female	32	28	87.50 (71.93, 95.03)
	male	7	1	14.29 (0.73, 51.31)
	total	39	29	74.36 (58.92, 85.43)

**Table 2:** The polymerase chain reactions (PCR) diagnose of *Theileria* in different hosts.

Hosts	Tick types	No.captured	No.PCR positive	Positivity rate (95% CI, %)
Cattle	female	46	40	86.96 (74.33, 93.88)
	male	14	2	14.29 (2.54, 39.94)
	total	60	42	70.00 (57.49, 80.10)
Sheep	female	74	64	86.49 (76.88, 92.49)
	male	28	3	10.71 (3.71, 27.20)
	total	102	67	65.69 (56.05, 74.18)

### 3.3 Identifications of *Theileria* species and phylogenic analysis

According to BLAST analysis, the 30 valid *Theileria* sequences obtained from sheep-infesting tick all matched *T. luwenshuni*. The obtained sequences showed 99.7–100% identity to the *T. luwenshuni* type strain sequences in NCBI GenBank across the entire sequenced region. A total of 11 successfully sequenced fragments from cattle-infesting ticks exhibited 99% homology with *T. orientalis*, *T. sergenti* and *T. sinensis* based on BLAST alignment. To achieve species-specific identification of *Theileria* in cattle-infesting ticks, we conducted further amplification using the *MPSP* gene of *T. orientalis* and *T. sinensis*. Based on the representative nucleic acid image results in Fig. S2A, it can be observed that the *MPSP* gene amplification product (744 bp) exhibits significantly more non-specific bands compared to the *18S rRNA* gene amplification product. Notably, PCR screening targeting the *T. orientalis MPSP* gene revealed 63.33% positivity, compared to 11.67% for *T. sinensis* (Fig. S2B). Eleven *MPSP* gene sequencing were conducted using 11 specimens with positive results from earlier *16S rRNA* sequencing, and the results showed 99.5% sequence identity with *T. orientalis*.

**Fig. 2:**
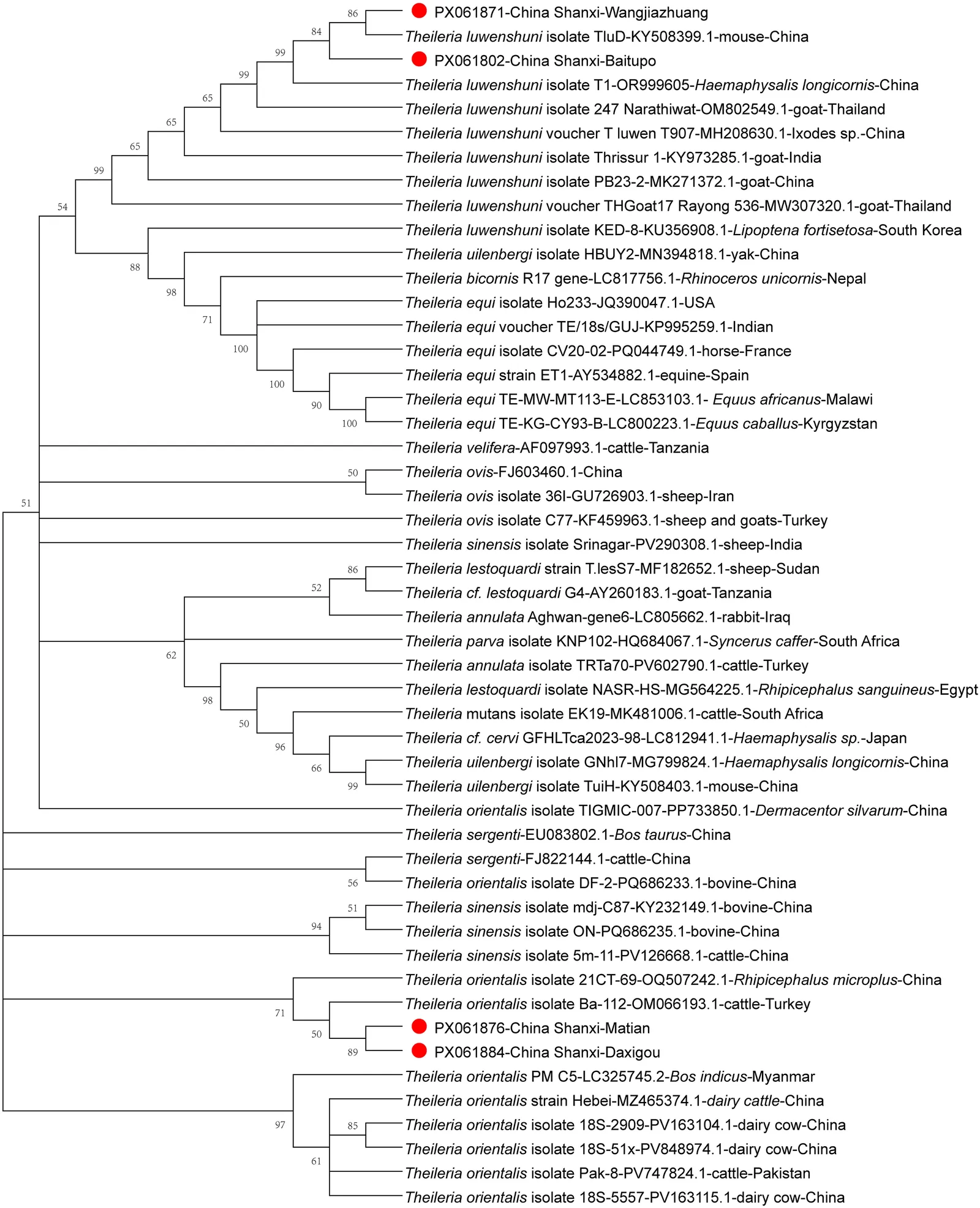
Phylogenetic analysis of *Theileria* spp. based on 18S rRNA gene sequences. The bootstrap value of phylogenetic tree is 500. Representative sequences from the four sampling sites in this study are indicated by black dots. Each entry is annotated with the corresponding *Theileria* species name, NCBI accession number, and geographic origin.

After assembly and quality control, the *18S rRNA* sequences of *Theileria* were submitted to the NCBI GenBank (accession no. PX061802 to PX061826 for *T. luwenshuni* isolates in Baitupo village, no. PX061871 to PX061875 for *T. luwenshuni* isolates in Wangjiazhuang Village, no. PX061876 to PX061883 for *T. orientalis* isolates in Matian Town, no. PX061884 to PX061886 for *T. orientalis* isolates in Daxigou Village). To elucidate the phylogenetic relationships of the *T. luwenshuni* and *T. orientalis* sequences obtained in this study, we performed phylogenetic analysis using the Neighbor-Joining method in MEGA-X. The analysis included a set of 46 representative *Theileria 18S rRNA* gene sequences retrieved from GenBank (Fig. 2). The phylogenetic tree revealed that the *T. luwenshuni* isolates in this study clustered with the mouse-derived *T. luwenshuni* isolate TluD (GenBank accession no. KY508399.1) from Kunming, China. Additionally, these isolates shared a tight evolutionary affinity with the Changzhi, Shanxi strain from our prior work (accession No. OR999605), indicating strong phylogenetic relatedness among Shanxi-derived *T. luwenshuni* strains. *T. orientalis* obtained in this study showed a strong phylogenetic relationship with cattle-derived isolates from Turkey (accession no. OM066193.1) and Hunan Province, China (accession no. OQ507242.1).

### 3.4 Phylogeographic network of *Theileria* strains

**Fig. 3:**
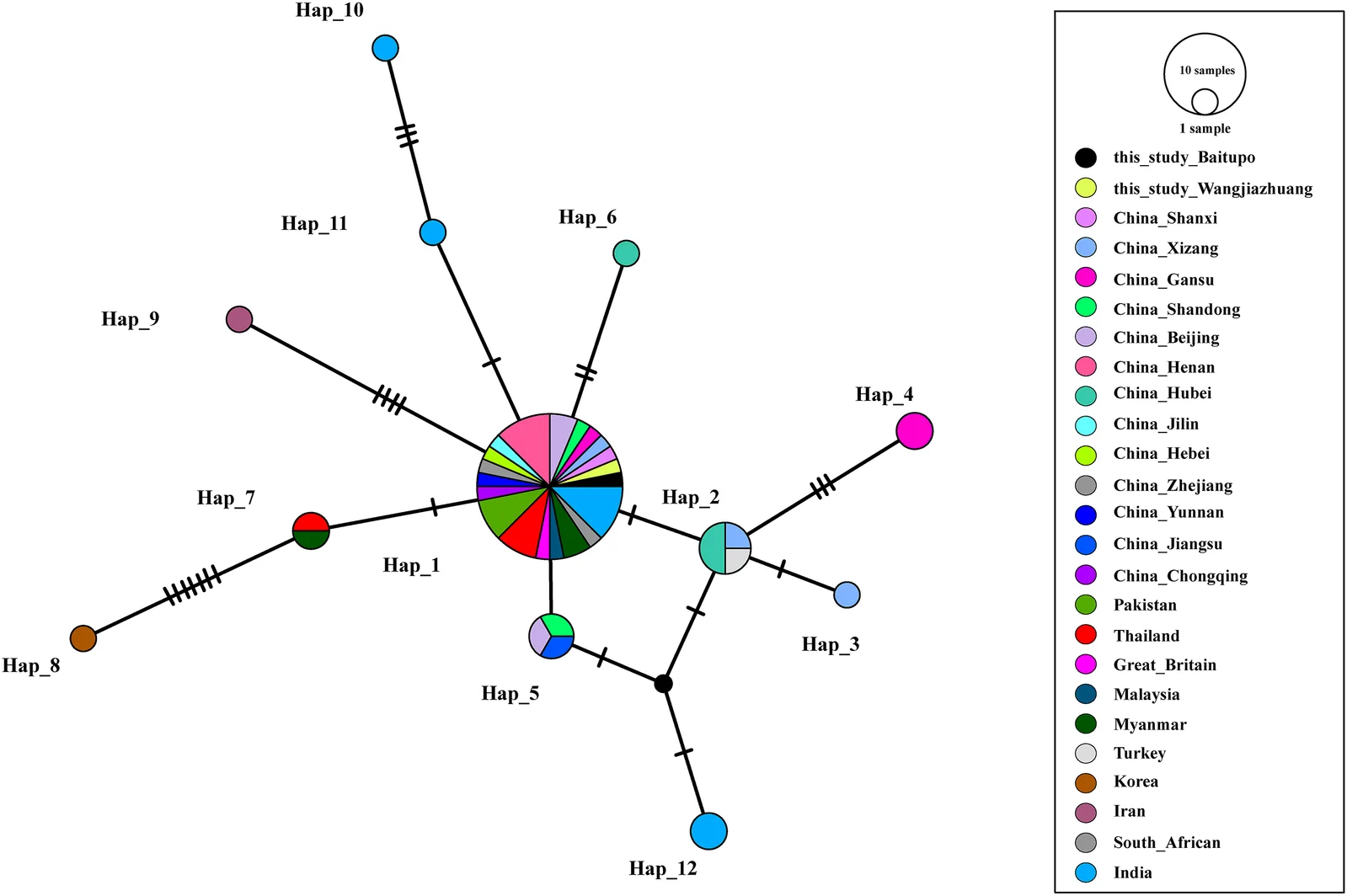
Phylogeographic network of *Theileria luwenshuni 18S rRNA* haplotypes (Median-Joining algorithm). Each color corresponds to a distinct geographic location.

Typical sequences of *T. luwenshuni* were selected from NCBI and subjected to haplotype evolutionary analysis along with the sequences obtained in this study. A total of 12 haplotypes were identified (*n* = 51, S = 20, h = 12, *π* = 0.01066, *κ* = 1.631, Hd = 0.602 ± 0.00631) (Fig. 3), and their corresponding hosts and sites were listed in Table 3. The haplotype network based on the Minimum spanning network method revealed the following: haplotype (Hap) 1 is the core haplotype, directly connected to multiple low-frequency Hap2, 5, 6, 7, 9, 11 via 1–4 mutational steps. High connectivity and phylogenetic position suggest Hap1 likely represents the ancestral haplotype, with isolates from China (*n* = 17) and other countries (*n* = 15) included. All *T. luwenshuni* isolates from this study shared identical full-length sequences. A single representative sequence from each sampling location was subjected to haplotype reconstruction, and both fell into Hap1. In addition, it should be noted that as shown in Table 3, abundant tick-derived *T. luwenshuni* isolates (KC580652.1, KJ715169.1, MH208630.1) from disparate geographical regions were uniformly assigned to haplotype 1, consistent with the Shanxi-derived strains.

**Table 3:** The haplotypes of *Theileria luwenshuni* involved in Fig. 3.

Haplotype	Acc. no	Host	Site
Hap1	PX061802	Sheep	China-Shanxi
Hap1	PX061871	Sheep	China-Shanxi
Hap1	OR999605	Sheep	China-Shanxi
Hap1	KU518032.1	Cattle	China -Xizang
Hap1	KU554730.1	Bactrian camel	China-Gansu
Hap1	KC580652.1	Tick	China-Beijing
Hap1	KC429038.1	Goat	China Beijing
Hap1	KJ715169.1	Tick	China-Henan
Hap1	KJ715193.1	Sheep	China-Henan
Hap1	KJ715184.1	Hedgehog	China-Henan
Hap1	JX469519.1	Sheep	China-Henan
Hap1	KC414097.1	Sheep	China-Jilin
Hap1	KJ806992.1	Cattle	China-Hebei
Hap1	KJ850935.1	Sheep	China-Shandong
Hap1	JN676988.1	Sheep	China-Zhejiang
Hap1	MH208630.1	Ixodes	China-Yunnan
Hap1	KC735147.1	Ruminant	China-Chongqing
Hap1	PQ001943.1	Sheep	Pakistan
Hap1	PP328914.1	Sheep	Pakistan
Hap1	PQ001989.1	–	Pakistan
Hap1	OM802549.1	Goat	Thailand
Hap1	OM802548.1	Goat	Thailand
Hap1	MW307319.1	Goat	Thailand
Hap1	KU234526.1	Sheep	Great Britain
Hap1	LR130184.1	*Haemaphysalis bispinosa*	Malaysia
Hap1	LC326010.2	Goat	Myanmar
Hap1	LC326009.2	Goat	Myanmar
Hap1	KU870897.1	Giraffe	South Africa
Hap1	PV283097.1	Sheep	India
Hap1	PQ305665.1	Goat	India
Hap1	KY973285.1	Goat	India
Hap1	MG694565.1	Goat	India
Hap2	MN394815.1	Yak	China-Xizang
Hap2	JX469529.1	Sheep	China-Hubei
Hap2	KC735162.1	Sheep	China-Hubei
Hap2	KY283963.1	Goat	Turkey
Hap3	MN394814.1	Yak	China–Xizang
Hap4	KP407015.1	Roe deer	China-Gansu
Hap4	KP407013.1	Sika deer	China-Gansu
Hap5	KF170834.1	Ixodes	China-Shandong
Hap5	KC769997.1	Sheep	China-Beijing
Hap5	KM016463.1	Goat	China-Jiangsu
Hap6	KC735163.1	Sheep	China-Hubei
Hap7	MW307320.1	Goat	Thailand
Hap7	LC602484.1	Dog	Myanmar
Hap8	KU356907.1	*Lipoptena fortisetosa*	Korea
Hap9	KU507510.1	Dog	Iran
Hap10	PV290749.1	Sheep	India
Hap11	MZ573171.1	Sheep	India
Hap12	MH764585.1	Sheep	India
Hap12	MG855929.1	Sheep	India

**Fig. 4:**
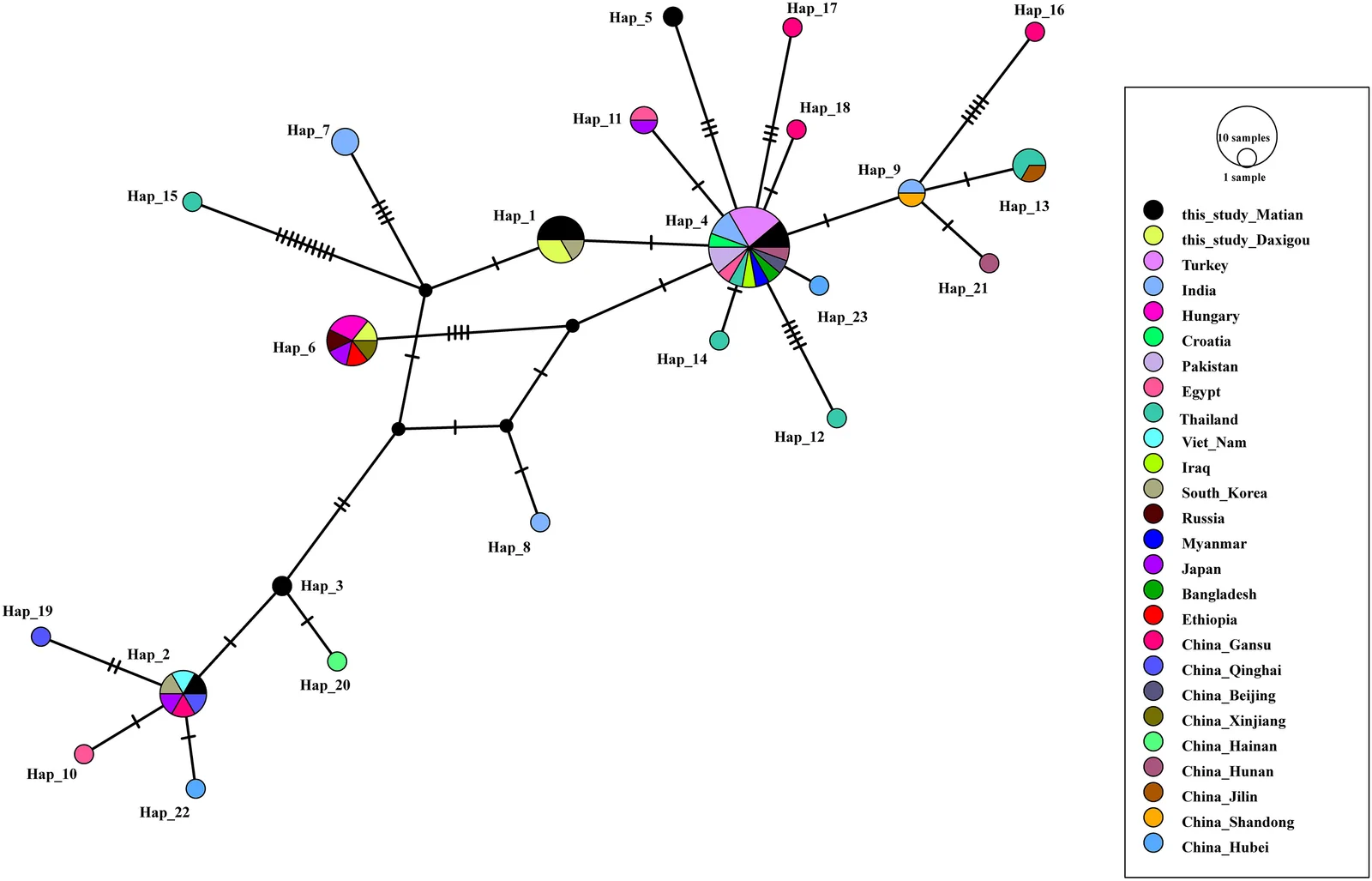
Phylogeographic network of *Theileria orientalis 18S rRNA* haplotypes (Median-Joining algorithm). Each color corresponds to a distinct geographic location.

The sequences of *T. orientalis* obtained in this study, along with the typical *T. orientalis* sequences collected from NCBI, were classified into 23 haplotypes (*n* = 62, S = 44, h = 23, *π* = 0.01149, *κ* = 4.756, Hd = 0.888 ± 0.00083) (Fig. 4, Table 4). Haplotype network analysis revealed that *T. orientalis* Hap4 is the core haplotype, and eight haplotypes (Hap1, 5, 9, 11, 12, 14, 17, 18) are directly derived from the core haplotype through minor allelic variations. Through successive evolutionary mutations diverging from the core haplotype, haplotype 2 and 6 have emerged as pivotal genetic variants, ultimately establishing the second-largest haplotype network. The *T. orientalis* isolates from Matian town were classified into 5 haplotypes (Hap1, 2, 3, 4, 5), while those from Daxigou Village were assigned to 2 haplotypes (Hap1, 6). Within the core haplotype, Chinese isolates accounted for a relatively small proportion, primarily originating from Beijing, Shanxi, and Hubei in China.

**Table 4:** The haplotype of *Theileria orientalis* involved in the Fig. 4.

Haplotype	Acc. no	Host	Site
Hap1	PX061876	Cattle	China-Shanxi-Matian
Hap1	PX061877	Cattle	China-Shanxi-Matian
Hap1	PX061881	Cattle	China-Shanxi-Matian
Hap1	PX061884	Cattle	China-Shanxi-Daxigou
Hap1	PX061885	Cattle	China-Shanxi- Daxigou
Hap1	MT052400.1	*Haemaphysalis longicornis*	South Korea
Hap2	PX061878	Cattle	China-Shanxi-Matian
Hap2	PP949827.1	Horse	Viet Nam
Hap2	MT889733.1	Cattle	South Korea
Hap2	XR696411.1	–	Japan
Hap2	MZ465374.1	Dairy cattle	China:Gansu
Hap2	PV163115.1	Dairy cow	China: Qinghai
Hap3	PX061879	Cattle	China-Shanxi-Matian
Hap4	PX061880	Cattle	China-Shanxi-Matian
Hap4	PX061882	Cattle	China-Shanxi-Matian
Hap4	OR211420.1	Cattle	Turkey
Hap4	OR211416.1	Cattle	Turkey
Hap4	OM066208.1	Cattle	Turkey
Hap4	OM066199.1	Cattle	Turkey
Hap4	OR068053.1	Buffalo	India
Hap4	OR068051.1	Buffalo	India
Hap4	PV613525.1	*Bos taurus*	Croatia
Hap4	MG599099.1	Cattle	Pakistan
Hap4	PP828601.1	Ruminant	Pakistan
Hap4	PP843606.1	–	Egypt
Hap4	PP380182.1	Bairy cattle	Thailand
Hap4	MH503884.1	Buffalo	Iraq
Hap4	LC602478.1	*Canis familiaris*	Myanmar
Hap4	MF576178.1	Bovine	Bangladesh
Hap4	PQ207062.1	Tick	China:Beijing
Hap4	OQ507250.1	*Rhipicephalus microplus*	China: Hunan
Hap5	PX061883	Cattle	China-Shanxi-Matian
Hap6	PX061886	Cattle	China-Shanxi- Daxigou
Hap6	MN611176.1	*Stomoxys calcitrans*	Hungary
Hap6	KU958549.1	*Ixodes simplex*	Hungary
Hap6	MW774588.1	Cattle	Russia
Hap6	AB668373.1	*Bos taurus*	Japan
Hap6	KJ941111.1	*Rhipicephalus decoloratus*	Ethiopia
Hap6	PQ686233.1	Bovine	China:Xinjiang
Hap7	KM609973.1	Buffalo	India
Hap7	MF458486.1	*Bos taurus*	India
Hap8	OR068052.1	Buffalo	India
Hap9	MT768053.1	*Rhipicephalus microplus*	India
Hap9	MH208642.1	*Rhipicephalus microplus*	China: Shandong
Hap10	PP937591.1	*Rhipicephalus annulatus*	Egypt
Hap11	OQ236626.1	–	Egypt
Hap11	LC576821.1	*Bos taurus*	Myanmar
Hap12	PP380181.1	Beef cattle	Thailand
Hap13	KC414103.1	Sheep	Thailand
Hap13	OM802550.1	Goat	Thailand
Hap13	OQ195761.1	*Haemaphysalis longicornis*	China: Jilin
Hap14	PP380178.1	Beef cattle	Thailand
Hap15	MG757650.1	–	Thailand
Hap16	MG599089.1	Cattle	China: Gansu
Hap17	MG799816.1	Cattle	China: Gansu
Hap18	KU363043.1	Cattle	China: Gansu
Hap19	PV848974.1	Dairy cow	China: Qinghai
Hap20	OR467408.1	*Rhipicephalus microplus*	China: Beijing
Hap20	OR837005.1	*Rhipicephalus microplus*	China: Hainan
Hap21	OQ507243.1	*Rhipicephalus microplus*	China: Hunan
Hap22	HM538266.1	–	China: Hubei
Hap23	HM538221.1	Cattle	China: Hubei

## 4 Discussion

Driven by human activity expansion and climate change, the geographic distribution of tick-borne pathogens is continuously broadening. Emerging and re-emerging tick-borne diseases have shown a significant global increase in incidence, posing serious threats to public health systems, livestock industries, and socioeconomic stability (Lawrence et al., 2017; Ebani et al., 2025). Understanding the biological characteristics, transmission dynamics, and pathogenesis of tick-borne pathogens is critical for devising targeted and effective disease control strategies.

*Theileria* is a critical tick-borne haemoprotozoan parasite, and infects a wide spectrum of mammalian hosts, including domestic animals such as cattle, sheep, and horses. These vertebrates act as parasite reservoirs, whereas ticks function as key biological vectors. They transmit *Theileria* among hosts during blood feeding and sustain the parasite's complete life cycle (Tonui et al., 2018). Infections with *Theileria* can persist asymptomatically in livestock or cause severe disease (Pitel et al., 2010). These infections significantly impair livestock productivity, thereby posing a serious threat to agricultural sustainability. Our study results indicate that the ixodid tick in the surveyed area serves as potential vectors for *Theileria*, suggesting that local livestock and wildlife could be at risk of theileriosis.

*T. luwenshuni* is primarily transmitted by various tick species of the family Ixodidae, including: *Haemaphysalis longicornis*, *Rhipicephalus microplus*, *Hyalomma asiaticum* (Li et al., 2015). Of these tick species, *H. longicornis* represents the key vector of *T. luwenshuni*, mediating transmission of this protozoan to susceptible hosts. This ixodid tick exhibits a broad geographic distribution, thriving in temperate and subtropical regions, which aligns with the endemic areas of *T. luwenshuni* infections (Yin et al., 2007). Moreover, some reports have indicated a relatively high infection rate of *T. luwenshuni* in sheep or cattle flocks (Chatanga et al., 2022; Sandamali et al., 2025), imposing a substantial burden on livestock health.

*T. orientalis* is primarily transmitted through hematophagous vectors, particularly *Haemaphysalis*, *Rhipicephalus, Stomoxys*, and *Haematobia* (Manjunatha Reddy et al., 2025). It is a causative agent of bovine oriental theileriosis and predominately endemic across Asia and Australia. Cattle and buffalo are highly susceptible of *T. orientalis*, with occasional transmission to sheep (Wang et al., 2017; Chen et al., 2022). In this study, a high infection rate of *T. orientalis* was detected in cattle-infesting ticks using partial *18S rRNA* gene-targeted PCR. The *T. orientalis MPSP* gene is also frequently employed in molecular detection assays, but gel image analysis and sequencing chromatograms of nucleic acid amplicons indicates that detection specificity is comparatively lower than that of the *18S rRNA* gene (Fig. S2). Based on BLAST analysis, we found that The *T. orientalis* strains isolated from cattle-infecting ticks in this study belongs to the *T.orientalis* complex, which includes *T.sergenti*, *T.buffeli* and *T.orientalis* in GenBank. Phylogenetic tree analysis revealed that these sequences clustered within the *T.orientalis* clade, and haplotype analysis further elucidated the genetic mutation and evolutionary history.

In this study, the average infection rate of *T. luwenshuni* is up to 65.30% in the central of Shanxi Province, which is higher than those detected in Changzhi, southeastern Shanxi Province (Cui et al., 2024). The high prevalence observed in central Shanxi province could result from sampling bias or underlying regional ecological differences. Meanwhile, we found that female ticks had a higher infection rate than males (86.67% vs 11.90%, *χ*^2^ = 30.446, *P* < 0.001), which is consistent with our previous findings (Cui et al., 2024). This may due to the smaller body size of male ticks, leading to lower genomic DNA extraction yields, while the richment of erythrocytes of female ticks. The infection rate of *Theileria* in cattle-infesting ticks was slight higher than that in sheep-infesting ticks, which is consistent with most reported literature (Qin et al., 2016; Chen et al., 2022). This is likely due to cattle maintaining a more stable body temperature near the parasite's optimal 37 °C, whereas sheep exhibit greater diurnal fluctuations. Of course, it may also be associated with multiple factors, including animal physiology, rearing environment, and parasite ecological adaptation. This study detected only one *Theileria* species-*T. luwenshuni*, in contrast to the greater diversity found in other Chinese regions, which may be attributed to ecological factors, sampling methods, or variations in the sensitivity of detection techniques. Further research should incorporate larger sample sizes and multi-seasonal sampling to validate the prevalence and dynamic changes of *Theileria* in tick populations.

Collectively, modern molecular biology techniques have enhanced the sensitivity and specificity of detecting *Theileria* from trace tick tissue specimens. Such methodologies encompass polymerase chain reaction (PCR), real-time quantitative PCR (qPCR), high-throughput sequencing, and loop-mediated isothermal amplification (LAMP), clustered regularly interspaced short palindromic repeats (CRISPR) (Henson et al., 2012; Gad et al., 2023; Prathyusha et al., 2025). In the present study, species-level identification of *Theileria* was performed via phylogenetic analysis of the *18S rRNA* gene. This marker exhibits high sequence conservation and thus allows preliminary screening at the species level. Nevertheless, its limited sequence divergence fails to discriminate closely related *Theileria* species and genotypes (Bawm et al., 2021; Kumar et al., 2022). Accordingly, the *18S rRNA* gene cannot guarantee accurate taxonomic delineation of complex mixed infections or novel *Theileria* species, which constitutes one limitation of the present study.

Besides, there are also many tough obstacles to the detection and prevention of theileriosis, attributable to the genetic diversity of *Theileria*, geographically varied endemic profiles, and complex host–pathogen–vector interaction networks. The geographic evolutionary map indicates that *T. luwenshuni* isolates exhibits relatively limited and conserved mutations, and *T.orientalis* exhibit a certain degree of genetic diversity (Fig. 3, Fig. 4). These biological complexities are further compounded by anthropogenic and environmental drivers—including climate change, intensive livestock farming, and transboundary animal movements—that collectively amplify arthropod-borne pathogen transmission risks (Zhang et al., 2025). Meanwhile, recent drastic biodiversity loss and shifts in vegetation structure have heightened the risk of tick-borne diseases (Diuk-Wasser et al., 2021). Consequently, an integrated framework for pathogen identification and disease prevention and control needs to integrate multilocus phylogenetic analysis, high-throughput sequencing technologies, and ecological risk modeling.

Notably, surveys of ticks collected from animal hosts to quantify tick abundance and pathogen transmission risk are prone to bias, stemming from divergent tick host preferences and uneven host susceptibility to different tick species (Gray et al., 2021). Furthermore, the genomic DNA extraction approach using intact whole ticks in the present study also bears inherent limitations. When using engorged ticks for pathogen screening, it may be impossible to distinguish whether the detected pathogens originate from infections within the tick's own tissues or residual host blood acquired during blood feeding. This also prevents us from clarifying whether tick infection of *Theileria* arises from active vector transmission or passive contamination. Accordingly, these assays cannot directly reflect the true transmission risk and clinical hazard posed by tick-borne pathogens. In follow-up studies, detection of host animal blood samples can be supplemented, and tick salivary glands can be isolated for separate sequencing, so as to clearly determine whether the pathogens are intrinsically carried by the tick vectors or derived from infected hosts.

## 5 Conclusion

Through *18S rRNA* gene amplification and sequencing, this study revealed a high prevalence of *T. luwenshuni* and *T. orientalis* detected in *H. longicornis* in central and southern Shanxi Province, China. This constitutes the first molecular evidence of *Theileria* spp. infection in this region, extending the known geographic distribution of these pathogens. Haplotype network illustrates haplotype mutation trends and the regional evolutionary pattern for *T. luwenshuni* and *T. orientalis*. In Shanxi Province, *T. luwenshuni* exhibits evolutionary stasis with only a single haplotype, whereas *T. orientalis* demonstrates multiple haplotypes and higher evolutionary diversity. Despite limitations, our findings highlight the necessity of long-term surveillance for tick-borne pathogens and lay the foundation for future research directions. A deeper understanding of the biological characteristics and pathogenic mechanisms of *T. luwenshuni* and *T. orientalis* is crucial for developing effective control strategies.

## CRediT authorship contribution statement

**Jia Cui:** Writing – review & editing, Writing – original draft, Methodology, Investigation. **Fengping Wang:** Writing – review & editing, Investigation. **Huaxiang Rao:** Investigation, Data curation. **Bosi Dang:** Methodology. **Yuyao Dou:** Methodology. **Yiping Liu:** Methodology. **Jingrong Niu:** Methodology. **Liqing Wang:** Data curation. **Changxin Wu:** Writing – review & editing, Supervision, Conceptualization. **Juan Yu:** Writing – review & editing, Supervision, Resources, Conceptualization.

## Funding

The present work was supported by Fundamental Research Program of Shanxi Province of China (202303021221180, 202303021212143), College Students' Innovative Entrepreneurial Training Plan Program of Shanxi Province of China (20251119, 20251177).

## Declaration of competing interest

The authors declare no competing interests.

## Data Availability

The sequencing data that support this study are openly available in the NCBI database under accession number PX061802 to PX061826, and PX061871 to PX061886. All data supporting the findings of this study are available within the paper and its Supplementary Information.
