# Supplementary material for: Prevalence and molecular detection of Theileria in ixodid ticks in central and southern Shanxi, China
Source: Parasite Epidemiol Control. 2026 Aug 14;34:e00533. doi: 10.1016/j.parepi.2026.e00533 (PMC13520614; doi:10.1016/j.parepi.2026.e00533)
Supplement: Supplementary file 1 [file mmc1.docx]

**Supplementary Figure Legends**

Figure S1 Agarose gel image of *18S rRNA* gene amplification of *Theileria* spp. in tick samples. Lane M: DL2000 DNA ladder; Lane 1-24: PCR product of partial tick samples

Figure S2 Agarose gel images of *MPSP* gene amplification of *Theileria* spp*.* in tick samples. (A) *MPSP* gene amplification of *Theileria orientalis*, (B) *MPSP* gene amplification of *Theileria sinensis*. DNA Lane M: DL2000 DNA ladder; DNA Lane 1-24: PCR product of partial tick samples

Figure S1 Agarose gel image of *18S rRNA* gene amplification of *Theileria* spp. in tick samples. Lane M: DL2000 DNA ladder; Lane 1-24: PCR product of partial tick samples

Figure S2 Agarose gel images of *MPSP* gene amplification of *Theileria* spp*.* in tick samples. (A) *MPSP* gene amplification of *Theileria orientalis*, (B) *MPSP* gene amplification of *Theileria sinensis*. DNA Lane M: DL2000 DNA ladder; DNA Lane 1-24: PCR product of partial tick samples

**Supplementary Table**

Table S1 The polymerase chain reactions (PCR) diagnose of *Theileria* spp. in female and male ticks

| Types | No.captured | No.PCR positive | Positivity rate (95% CI, %) |
| --- | --- | --- | --- |
| female ticks | 120 | 104 | 86.67（79.44-91.62） |
| male ticks | 42 | 5 | 11.90（5.19-25.00） |
